# Supplementary material for: Rai1 frees mice from the repression of active wake behaviors by light
Source: eLife. 2017 May 26;6:e23292. doi: 10.7554/eLife.23292 (PMC5464769; doi:10.7554/eLife.23292)
Supplement: Figure 2—source data 1. — n = 4/genotype. DOI: http://dx.doi.org/10.7554/eLife.23292.006 [file elife-23292-fig2-data1.docx]

| [/24h] | *Rai1^+/+^* | *Rai1^+/-^* | ***P*** |
| --- | --- | --- | --- |
| **Total wake [h]** | 14.71 ± 0.22 | 14.08 ± 0.10 | *0.06* |
| **TDW [h]** | 5.17 ± 0.26 | 3.66 ± 0.49 | *0.04* |
| **NREM sleep [h]** | 7.89 ± 0.26 | 8.63 ± 0.15 | *0.06* |
| **REM sleep [h]** | 1.40 ± 0.06 | 1.30 ± 0.08 | *0.30* |
